# An improved lightweight high-resolution network based on multi-dimensional weighting for human pose estimation

**DOI:** 10.1038/s41598-023-33938-x

**Published:** 2023-05-04

**Authors:** Lei Zhang, Jia-Chun Zheng, Shi-Jia Zhao

**Affiliations:** School of Ocean Information Engineering, Jimei University, Xiamen, 361021 People’s Republic of China

**Keywords:** Computer science, Computational science

## Abstract

Human pose estimation is one of the key technologies in action recognition, motion analysis, human–computer interaction, animation generation etc. How to improve its performance has become a current research hotspot. Lite-HRNet establishes long range connections between keypoints and exhibits good performance in human pose estimation tasks. However, the scale of this method to extract features is relatively single and lacks sufficient information interaction channels. To solve this problem, we propose an improved lightweight high-resolution network based on multi-dimensional weighting, named MDW-HRNet, which is implemented by the following aspects: first, we propose global context modeling, which can learn multi-channel and multi-scale resolution information weights. Second, a cross-channel dynamic convolution module is designed, it performs inter-channel attention aggregation between dynamic and parallel kernels, replacing the basic convolution module. These make the network capable of channel weighting, spatial weighting and convolution weighting. At the same time, we simplify the network structure to perform information exchange and information compensation between high-resolution modules while ensuring speed and accuracy. Experimental results show that our method achieves good performance on both COCO and MPII human pose estimation datasets, and its accuracy surpasses mainstream lightweight pose estimation networks without increasing computational complexity.

## Introduction

As a basic task of motion recognition, human pose estimation is widely used in emerging fields such as action recognition and behavior analysis. The core of this task is to locate and classify the keypoints of human in the image^1^, but it is limited by factors such as occlusion, light, environmental changes, and human appearance changes, which brings certain challenges. An early solution for pose estimation is to regress the coordinate points, and more and more researchers are now using convolutional neural networks to deal with this task.

Recent studies have shown that maintaining high-resolution representations of heatmaps is an important means of ensuring accuracy in pose estimation tasks. HRNet^2–4^ can be seen as a representative method among them. However, this will bring relatively high computing requirements. When the model accuracy is sufficient for application, people begin to pursue more efficient pose estimation networks. Existing lightweight networks are mainly designed from two aspects, the first is to obtain modular depthwise separable convolutions from efficient networks such as MobileNet^5,6^ and ShuffleNet^7,8^, then combine them with high-resolution models to form the network backbone. The second is to weight the channel information to establish a long-distance connection, which is also the basic design idea of Lite-HRNet^9^. Lite-HRNet shows that an efficient convolution module can replace the basic 3 × 3 convolution and 1 × 1 convolution for feature extraction, but the static and fixed network structure limits the performance to a certain extent. From a dynamic point of view, Dite-HRNet^10^ proposes that using different design parameters at different depths and widths of the network will bring certain performance gains, and design a dynamic network structure accordingly. Our work borrows the idea of dynamic parameters from Dite-HRNet and focuses on further streamlining the network and improving performance.

From recent studies^1,11–14^, parallel and multi-dimensional computation can lead to improvements in human pose estimation, which can be achieved by convolution and channel weighting. The existing weighting methods^15–17^ are mostly based on SE Attention^18^ and use global average pooling for preprocessing. However, this approach will bring some performance loss to the fine-grained task of pose estimation. Therefore, how to design an efficient and accurate information extraction method is a problem we must face.

To address this issue, we propose an improved multi-dimensional weighted high-resolution network named MDW-HRnet, we designed a convolution module with multi-dimensional weighting and a new information exchange module to further improve the performance. Specifically, the multi-dimensional weighted convolution module consists of contextual information modeling and cross-channel dynamic convolution, this makes the network capable of channel weighting, spatial weighting and convolution weighting. Context information modeling is divided into channel branch and space branch. High-resolution features are always maintained in channel and space dimensions to reduce information compression caused by dimensionality reduction operations. At the same time, cross-channel dynamic convolution adopts one-dimensional convolution as the basic operation of cross-channel interaction, and conducts sufficient information exchange, which is used to replace normal convolution. Our information exchange module can perform multi-scale fusion with reduced computational requirements, and the above components constitute the basic components of our MDW-HRNet.

Our main contributions include:

1. We propose contextual information modeling, perform pixel-level weighting on the corresponding dimensions of the input tensor, perform sufficient compression and restoration, and always maintain high-resolution features.
2. We design cross-channel dynamic convolution, which uses one-dimensional convolution to form the exchange part of dynamic attention, assists in cross-channel information extraction, and is used to replace the basic convolution module.
3. Perform information exchange between high-resolution modules while ensuring accuracy and speed, make full use of the channel and space representation capabilities, and perform information exchange and information compensation within multiple resolution branches without increasing additional computing power requirements.

## Related work

### High resolution network

Early networks for pose estimation usually consist of a series of high-resolution to low-resolution subnetworks connected, and this structure causes a certain loss of accuracy for fine pixel-level tasks. The high-resolution network^2–4^ and its derived structures can maintain high-resolution representations throughout the feature extraction process, gradually generate low-resolution subnetworks from high-resolution subnetworks, and fully exchange information between different resolution subnetworks. Lite-HRNet inherits this structural property and replaces expensive bottleneck convolutional layers with conditional channel weighting composed of attention. Dite-HRNet proposes that using different network parameters at different depths and widths of the network will bring certain gains to performance, and adds a high-efficiency CNN module composed of Dynamic Kernel Aggregation to further connect the dependencies between the upper and lower environments. It is worth noting that the attention module adopted by Dynamic Kernel Aggregation in Dite-HRNet is highly coincident with its proposed Global Context Modeling, which will bring about repetitive information modeling to a certain extent. Our solution is to establish cross-channel dynamic convolution for replacement, and employ 1D convolution as a means of exchanging channel information.

### Efficient network structure

Depthwise separable convolution was proposed in MobileNet V1 and is widely used in lightweight network structures, which can compress computational costs while ensuring performance; MobileNet V2 introduces a reverse bottleneck layer, which increases the network width and improves accuracy and efficiency compared to traditional bottleneck blocks; ShuffleNetV2 proposes channel shuffling, which only performs convolution operations on half of the channels after separating channels, and enhances cross-channel information interaction through channel shuffling, which provides design ideas for lightweight networks; MixNet^19^ explored a collection of convolution kernels of different sizes, which became the basic idea of split convolution; Inspired by ResNet^20^, RSN^1^ performs feature fusion between multiple convolutional blocks stepwise and cumulatively; ConvNext^21^ proposed a large kernel convolution combined with an inverse bottleneck layer to reduce the computational complexity while increasing the accuracy.

### Dynamic convolution

CondConvolution^22^ breaks the traditional static convolution characteristics by changing the input calculation convolution kernel parameters, avoiding the disadvantage that all samples share a convolution kernel in the traditional static convolution, and only increases a small amount of calculation, which is used to replace ordinary convolution. Dynamic convolution^23^ uses input-based attention weights for dynamic aggregation, it shares output channels through parallel convolution kernels, at the same time, the attention is used to weight the convolution kernels, that is, the convolution kernels of the same input and output are aggregated through the attention dimension. ODConv^24^ utilizes a multi-dimensional attention mechanism with a parallel strategy to learn complementary attention for convolutional kernels along all four dimensions of the kernel space at any convolutional layer. Based on the above work, we propose cross-channel dynamic convolution, which performs local channel interaction through one-dimensional convolution while avoiding excessive dimensionality reduction operations.

### Information aggregation

NLNet^25^ considers that the response of a position is also the sum of the response weights of the feature maps inputted at all positions, thus linking the local to the global. GCNet^26^ establishes a unified long-range dependency for different locations of the network to strengthen the original features, which reduces a certain amount of computation compared to NLNet. DANet^27^ adopts a dual attention mechanism, adding parallel spatial pooling module and position pooling module to the network and fuses the output of the attention module to increase performance. PSA^28^ uses a polarization filtering mechanism to compress a certain dimension while maintaining high-resolution information in its orthogonal dimension, and uses nonlinearity to compensate for the loss of information, and achieves high performance in attitude estimation tasks. The implementation of GCM draws on the PSA method to a certain extent, which will be described in “Channel weighting” Section.

## Our method

### Network structure

#### Modeling

**Figure 1:**
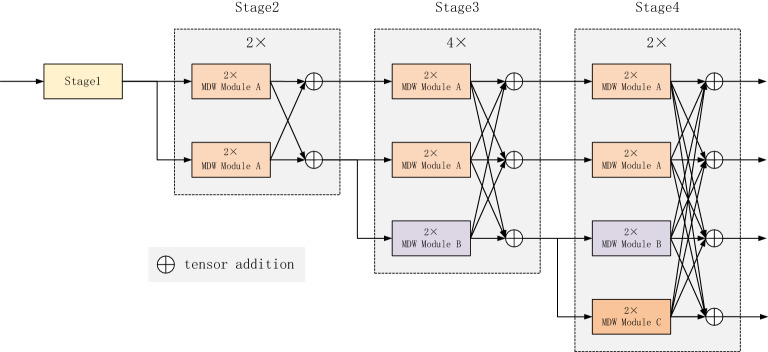
MDW-HRNet network structure.

The MDW-HRNet network structure is shown in Fig. 1, the network will maintain a high-resolution representation of the heatmap throughout, and incrementally add sub-networks from high to low resolution, each newly added sub-network has half the resolution and twice the width of the previous sub-network, and performs sufficient feature fusion between multi-resolution branches. The network is divided into four stages, each stage has up to four parallel branches. The first stage of the network is the stem module, which compresses the heat map resolution to an appropriate size while performing feature extraction and performs subsequent maintenance work. Each stage of the network is composed of multi-dimensional weighted convolution module and a feature fusion module. In each stage, repeated feature extraction and a feature fusion involving multi-resolution branches are performed. We designed MDW-HRNet-18 and MDW-HRNet-30 with reference to the high-resolution network for comparison. As shown in Table 1, the network depth and width of MDW-HRNet are comparable to Lite-HRNet and Dite-HRNet, and the computational consumption is also at the same level.

**Table 1:** Structure of MDW-HRNet.

Layer	Operator	Output size	output_channels	MDW-HRNet-18 module	MDW-HRNet-30 module
Image		256 × 192	3		
Stage1	3 × 3conv Shuffle block	64 × 48	32	1	1
Stage2	2*MDW_CM fusion_block	64 × 48,32 × 24	40,80	2	3
Stage3	2*MDW_CM fusion_block	64 × 48,32 × 24,16 × 12	40,80,160	4	8
Stage4	2*MDW_CM fusion_block	64 × 48,32 × 24,16 × 12,8 × 6	40,80,160,320	2	3

#### Multi-dimensional weighted convolution module

**Figure 2:**
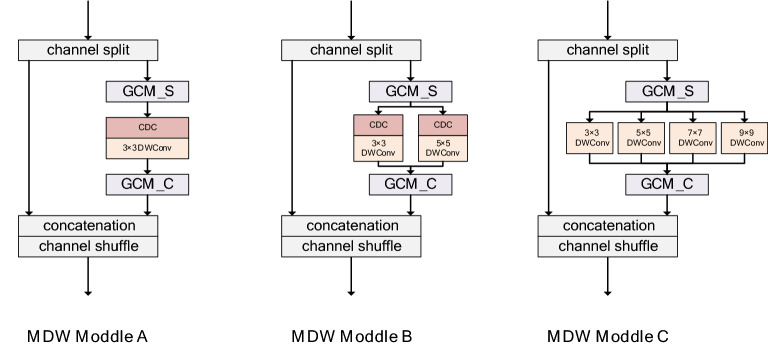
Multi-dimensional weighted convolution module.

Recent studies have shown that: a large receptive field is suitable for extracting feature relationships between different key points^29,30^, and fusing features of different spatial sizes helps the network to extract more refined local representations^1^. In order to obtain accurate spatial information, we design a multi-dimensional weighted convolution module and implement it into three specific types, namely module A, module B and module C, which are composed of global information modeling and dynamic convolution. this makes it capable of space weighting, channel weighting and convolution weighting. It will run through all stages of the network, and the specific structure is shown in Fig. 2. Module A is applied to high-resolution branches. Relying on cross-channel dynamic convolution and global context modeling, it has the ability of long-distance key point modeling and fast and accurate information extraction. Module A can establish the remote dependence between space and channels and conduct sufficient information exchange, which enables module A to extract more detailed local representation in high-resolution branches. Because only 3 is used × 3 Convolution core, module A is more efficient than module B and module C; In the third and fourth branches of the network, we use module B and module C to fuse the features between different receptive fields to obtain more accurate key point information, and at the same time, we further expand the receptive field in module C to obtain the correlation between different joints.

#### Sampling

In previous high-resolution networks, the downsampling operation was performed using 3 × 3 convolutions, which seems to be the undisputed first choice for the downsampling operation of the convolutional neural network, in the ViTPose^31^ and convnext^21^ networks, the settings of kernel_size = 2 and stride = 2 are used for downsampling. We follow this new setting, which is applied in the process of generating new branches in the high-resolution network and in the repeated multi-scale fusion process, and for the upsampling operation, we use bilinear interpolation for implementation. The number of channels will be aligned simultaneously during the downsampling operation. This will save some memory consumption.

For Fig. 1, the main body is divided into 4 stages, with 4 parallel branches, each stage will finally perform feature fusion, and use the branch with the highest resolution as the output. MDW module represents multi-dimensional weighted convolution module.

For Fig. 2, where GCM_S and GCM_C represent spatial context modeling and channel context modeling, and CDC represents cross-channel dynamic convolution.

For Table 1, the number of output channels corresponds to the size of the output heatmap. Stage1 contains a 3 × 3 convolution and a shuffle block, which simultaneously perform the operations of extracting features and increasing the network width. MDW_CM stands for multi-dimensional weighted convolution module, the contents in the fifth and sixth columns, the values in MDW-HRNet-18 module, represent the number of repetitions of corresponding operations in each layer.

### Global context modeling

The tensor dimensions of the input network can generally be expressed as C, H and W, which represent the number of channels of the feature tensor, and the height and width of the input tensor, denotes as ${\text{X}} \in {\text{R}}^{{{\text{C}} \times {\text{H}} \times {\text{W}}}}$, The work of context information modeling is to learn to filter out noise in high-resolution information, which can be expressed as (1)

1 $$ {\text{X}}^{\prime } = {\text{X}} \odot {\text{ W}}_{{\text{s}}} $$

where $W_{s}$ represents the long-distance dependent weight matrix, and $\odot$ represents the multiplication of two elements at the corresponding positions of two matrices. For the convenience of embedding, we set $X^{\prime}$ and $X$ to have the same number of channels.

We can abstract the process of computing ${\text{ W}}_{{\text{s}}}$ as follows:

1. Filtering: Compress the height and width features and channel features separately in a predetermined dimension, increase the nonlinear feature range through the softmax operation at the minimum channel feature, and fit the compressed features to the original dimensions to obtain long distance information.
2. Information fusion: The information is further integrated by 1 × 1 convolution. Add normalization and new nonlinear information. This process can be expressed as (2)
  2 $$ {\text{ W}}_{{\text{s}}} {\text{ = weight (reshape}}\left( {{\text{W}}_{{\text{c}}} \otimes {\text{W}}_{{1}} } \right){)} $$

Among them, ${\text{W}}_{{\text{c}}}$ stands for N × C × HW tensor dimension, ${\text{W}}_{{1}}$ stands for N × HW × 1 tensor dimension respectively, $\otimes$ stands for tensor multiplication, reshape($\cdot$) represents the dimension of the restored weight after compression, and weight($\cdot$) represents the information fusion operation: twice 1 × 1 Convolution. To further mine the performance, we define the context information modeling as two parts, the channel weighting and the space weighting, which will fully release the gain it brings in the corresponding dimension.

#### Channel weighting

**Figure 3:**
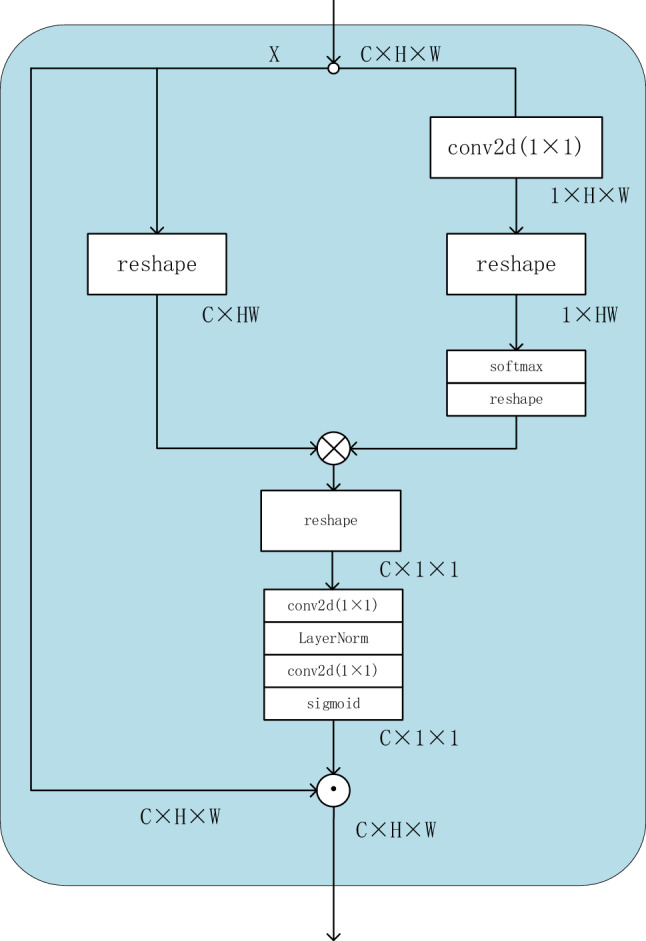
Structure of channel weighting.

The design of the Channel weighting is based on the idea of PSA to a certain extent, it will be used throughout the whole network, it always maintains the highest resolution in the process of compressing dimensions, while discarding global average pooling. Its weight matrix is composed of two compression matrices. One of the compression matrix dimensions is N × c × hw(the height of the characteristic diagram is h, the width is w, and the number of channels is c), another compression matrix dimension is N × hw × 1, and use softmax operation to increase nonlinear information, then merge the two compression matrices and perform information fusion. Due to the design of information filtering, the channel branch abandons the use of global average pooling to collect spatial information, and reduces the operation of compressing dimensions, further maintaining accuracy. Its structure is shown in Fig. 3.

For Fig. 3, x represents the input, where c, h and w represent the number of channels, height and width respectively.

#### Spatial weighting

MDW-HRNet has a total of 4 stages. In the nth stage, there are n parallel branches. The network width and resolution represented by each branch are different. The nth branch of any nth stage has the smallest heatmap resolution size and largest number of channels for that stage. We take advantage of this feature to properly compress the spatial information in the channel, perform dense modeling, and restore the resolution of the heatmap by upsampling after completing the information fusion, and perform a channel-by-channel weighting operation. In the nth stage, all heatmaps will be compressed to the minimum resolution size corresponding to this stage, as shown in (3)

3 $$ {\text{X}}_{{\text{i}}} \in {\text{R}}^{{{\text{c}} \times {\text{h}} \times {\text{w}}}} \to {\text{X}}_{{\text{i}}}^{^{\prime}} \in {\text{R}}^{{{\text{c}} \times {\text{h}}_{{\text{m}}} \times {\text{w}}_{{\text{m}}} }} $$

where i represents the input number of the $ith$ different resolution, and ${\text{H}}_{{\text{m}}} \times {\text{W}}_{{\text{m}}}$ represents the minimum resolution size. The channel information and spatial information are compressed and integrated separately in a predetermined dimension, and nonlinear information is added. The specific operation of spatial weighting is shown in (4):

4 $$ {\text{X}}_{{\text{s}}} = {\text{ X}}\left\{ {{\text{upsample}}\,\left( {{\text{sigmoid}}\,\left( {{\text{conv}}\,\left( {{\text{layernorm}}\,\left( {{\text{conv}}\left( {{\text{X}}_{i}^{^{\prime}} \in {\text{R}}^{{{\text{c}} \times {\text{h}}_{{\text{m}}} \times {\text{w}}_{{\text{m}}} }} } \right)} \right)} \right)} \right)} \right)} \right\} $$

Among them, conv represents the operation of conv2d (1 × 1), and layernorm is the normalization operation, and bilinear interpolation is used to achieve upsampling.

### Cross-channel dynamic convolution

Dynamic convolution has two basic elements, convolution kernel and weighting function for computing attention. It can be seen from the experiments of ODConv that the attention mechanism in dynamic convolution plays a key role in performance gain. After removing the attention of CondConv or DyConv, their gain to the network is almost 0. Therefore, designing an efficient convolutional attention mechanism is a necessary means to improve performance.

Traditional convolutional layers have a single, static kernel that is applied to all inputs. Dynamic convolution divides a single core into multiple cores and aggregates them linearly, and introduces an attention mechanism to associate convolution operations with input information. For the current mainstream dynamic convolutions such as CondConv, DyConv, and ODconv, they all use an attention structure similar to SE Attention. The difference is that CondConv uses Sigmoid and DyConv uses Softmax as the activation function to calculate the attention weight.

**Figure 4:**
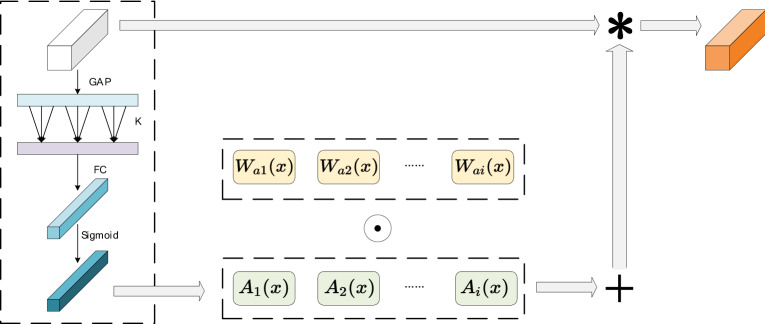
Cross-channel dynamic convolution. COCO pose estimation results removed.

The attention mechanisms embedded in the current mainstream dynamic convolution algorithms are mostly variants of SE Attention, or improve the attention module by combining more complex channel or spatial dependencies, which indirectly increases the complexity of the network. Inspired by ECANet^32^, we introduce channel information interaction in dynamic convolution while reducing dimensionality reduction operations to replace the traditional attention module, and achieve excellent performance. We will use one-dimensional convolution for cross-channel information interaction, as shown in Fig. 4. In order to increase performance, cross-channel dynamic convolution will employ multiple parallel convolution kernels that simultaneously accept the input of relevant attention and discard the bias term. Cross-channel dynamic convolution aims to capture possible local channel information interactions and embed them without reducing the network width during information extraction, and channels and weights correspond directly. Multiple parallel convolution kernels perform dynamic information aggregation for each input tensor through cross-channel attention, which has richer expressive ability than traditional convolution. Parallel cores share the same output channel after aggregation, controlling the computational cost. This module can be expressed as (5):

5 $$ {\text{A}}\left( {\text{X}} \right) = {\text{sigmoid}}\left( {{\text{Conv2d}}\left( {{3} \times {3}} \right)\left( {{\text{Conv1d}}\left( {\text{k}} \right)\left( {{\text{Gap}}\left( {\text{x}} \right)} \right)} \right)} \right) $$

Among them, A(X) is the attention weight of the convolution kernel, conv1d represents one-dimensional convolution, k = 3, Gap is the global average pooling operation, and one-dimensional convolution is calculated in the channel dimension, which will not affect the network depth.

Dynamic convolution layer can be expressed as a linear combination of n convolution cores, each convolution kernel will obtain learnable cross-channel attention weight, this makes the convolution operation have the information interaction of the input dimension. The dynamic convolution operation can be represented by formula (6):

6 $$ Y = \left[ {\mathop \sum \limits_{i}^{n} A_{i} \left( X \right)W_{i} } \right] \times X $$

where $$X^{\epsilon c \times h \times w}$$ and $$Y^{\epsilon c \times h \times w}$$ represent rthe characteristic diagrams of input and output respectively (the height of the characteristic diagram is h, the width is w, and the number of channels is c. For the convenience of calculation, it is assumed that the input and output have the same channel). Where $$A_{i} \left( X \right)$$ is the attention weight for the $$ith$$ convolution kernel, and $$ W_{i}$$ is the weight matrix for the $$ith$$ convolution kernel, and * represents convolution operation.

For Fig. 4, $W_{ai} \left( X \right)$ is the weight matrix corresponding to $A_{i} \left( X \right)$, $\odot$ represents the multiplication of two elements at the corresponding positions of two matrices, + represents the weight matrix of the aggregate convolution kernel, and * represents convolution operation. Global average pooling(GAP) is used to obtain the corresponding aggregation features, and the channel information is adjusted according to the one-dimensional convolution of size K to obtain the attention weight, and dynamic information aggregation is performed through multiple parallel convolution kernels.

## Experiments

### Implementation details

#### Datasets

The COCO dataset^33^ has more than 200 K images of human instances, each with 17 keypoint labels. We train our network on the train2017 dataset (containing 57 k images and 150 K human body instances) and evaluate the val2017 set according to the average precision (AP) and average recall (AR) scores based on object keypoint similarity (OKS) (contains 5 k images) and test-dev2017 set (contains 20 k images) for evaluation. To further validate our network, we also conduct experiments on the MPII Human Pose Dataset^34^, which contains about 25 k images with 40 k human instances and is scored by the head-normalized probability of correct keypoints (PCKh) to assess accuracy.

#### Training

The network is trained on a single RTX Titan with 32 samples. We use the Adam optimizer with an initial learning rate of 2${\text{e}}^{ - 3}$, which drops to 2${\text{e}}^{ - 4}$ and 2${\text{e}}^{ - 5}$ at the 170th and 200th epochs, respectively, for a total of 240 epochs of training. In terms of data processing, we scale all human detection boxes to a fixed 4:3 aspect ratio, and then use the detection boxes to crop the image, resized to 256 × 192 or 384 × 288 for COCO dataset. Set to 256 × 256 in the MPII dataset. Data augmentation operations were applied to all inputs, including setting a random rotation of 30°, random scale expansion by a factor of 0.25, and random flipping of the COCO and MPII datasets. Furthermore, a half-body transformation is performed on the COCO dataset.

#### Testing

We will conduct experiments in a two-stage top-down paradigm: first human detection by a person detector, followed by keypoint detection, where the person detector is provided by Simple Baseline^35^. We estimate the heatmap through a Gaussian filter and average the predicted heatmaps for the original and flipped images. Apply a quarter shift in the direction from the highest response peak to the second highest response peak to obtain the position of each keypoint. For the MPII dataset, we adopted the standard testing strategy from the provided Person box.

### Results

#### COCO val

The results of comparing our method with some mainstream methods are shown in Table 2. MDW-HRNet-18 is trained with an input size of 256 × 192 without pre-training, and finally achieves an AP score of 67.8, which is better than Other lightweight networks. Compared with Lite-HRNet, the highest 3AP score improvement is obtained with only 3% more parameters and 1% GFLOP consumption; The number of parameters required by MDW HRNet-18 and MDW-HRNet-30 is only 11.4% and 19% of MobileNetV2, but they have achieved 3.2AP and 4.8AP improvements, respectively. In the case of accepting an input of 384 × 288, MDW HRNet-30 achieves a score of 72.9 AP after 270 epochs of training, which is even close to traditional large networks. Compared with the state-of-the-art Dite-HRNet, MDW-HRNet also has certain advantages. With almost the same amount of parameters and computation, our network still achieves a 1.9 AP score improvement. These results benefit from the more lightweight structure of the network and our proposed new attention weighting method, and also prove that MDW-HRNet achieves a good balance between model accuracy and speed.

**Table 2:** Comparisons on the COCO val set.

Method	Backbone	Pretrain	Input size	#Params	GFLOPs	AP	${\text{AP}}^{{{50}}}$	${\text{AP}}^{75}$	${\text{AP}}^{{\text{M}}}$	${\text{AP}}^{{\text{L}}}$	AR
Large networks											
8-stage hourglass^32^	hourglass	N	256 × 192	25.1	14.3	66.9	–	–	–	–	–
CPN^30^	ResNet-50	Y	256 × 192	27.0	6.2	68.6	–	–	–	–	–
SimpleBaseline	ResNet-50	Y	256 × 192	34.0	8.9	70.4	88.6	78.3	67.1	77.2	76.3
HRNet	HRNet-W32	N	256 × 192	28.5	7.1	73.4	89.5	80.7	70.2	80.1	78.9
RSN	3 × RSN-50	N	256 × 192	15.0	4.9	74.9	90.0	82.3	71.6	81.2	81.2
Small networks											
Mobile NetV2 1 ×	MobileNetV2	N	256 × 192	9.6	1.4	64.6	87.4	72.3	61.1	71.2	70.7
ShuffleNetV2 1 ×	ShuffleNetV2	N	256 × 192	7.6	1.2	59.9	85.4	66.3	56.6	66.2	66.4
Small HRNet	HRNet-W18	N	256 × 192	1.3	0.5	55.2	83.7	62.4	52.3	61.0	62.1
Lite-HRNet	Lite HRNet-18	N	256 × 192	1.1	0.2	64.8	86.7	73.0	62.1	70.5	71.2
	Lite HRNet-30	N	256 × 192	1.8	0.3	67.2	88.0	75.0	64.3	73.1	73.3
Dite-HRNet	Dite HRNet-18	N	256 × 192	1.1	0.2	65.9	87.3	74.0	63.2	71.6	72.1
	Dite HRNet-30	N	256 × 192	1.8	0.3	68.3	88.2	76.2	65.5	74.1	74.2
MDW-HRNet (Ours)	MDW-HRNet-18	N	256 × 192	1.1	0.2	67.8	90.5	76.0	65.7	71.9	72.2
	MDW-HRNet-30	N	256 × 192	1.8	0.3	69.4	91.5	77.0	67.5	74.3	74.7
MobileNetV2 1 ×	MobileNetV2	N	384 × 288	9.6	3.3	67.3	87.9	74.3	62.8	74.7	72.9
ShuffleNetV2 1 ×	ShuffleNetV2	N	384 × 288	7.6	2.8	63.6	86.5	70.5	59.5	70.7	69.7
Small HRNet	HRNet-W18	N	384 × 288	1.3	1.2	56.0	83.8	63.0	52.4	62.6	62.6
Lite-HRNet	Lite HRNet-18	N	384 × 288	1.1	0.4	67.6	87.8	75.0	64.5	73.7	73.7
	Lite HRNet-30	N	384 × 288	1.8	0.7	70.4	88.7	77.7	67.5	76.3	76.2
Dite-HRNet	Dite HRNet-18	N	384 × 288	1.1	0.4	69.0	88.0	76.0	65.5	75.5	75.0
	Dite HRNet-30	N	384 × 288	1.8	0.7	71.5	88.9	78.2	68.2	77.7	77.2
MDW-HRNet(Ours)	MDW-HRNet-18	N	384 × 288	1.1	0.4	70.2	90.5	77.4	67.8	75.9	75.3
	MDW -HRNet-30	N	384 × 288	1.8	0.7	72.9	91.6	80.4	70.5	77.9	77.8

For Table 2, where pretrain indicates that the backbone network is pre-trained using the imageNet dataset. #Params and FLOPS are computed for the pose estimation network, excluding human detection and keypoint grouping.

#### COCO test-dev

The results of comparing our network with other methods are shown in Table 3. MDW-HRNet achieves the highest AP score of 70.9, which is significantly better than other small networks in terms of efficiency and accuracy. In the case of almost close to the computational consumption of Lite-HRNet, MDW-HRNet achieves a maximum improvement of 2.1 AP score. Compared with the large network HRNet, MDW-HRNet shows amazing computational performance, its params and FLOPs consumption is only 6.3% and 4.3% of HRNet, Yet it shows amazing results.

**Table 3:** Comparisons on the COCO test-dev set.

Method	Backbone	Input size	#params	GFLOPs	AP	${\text{AP}}^{50}$	${\text{AP}}^{75}$	${\text{AP}}^{{\text{M}}}$	${\text{AP}}^{{\text{L}}}$	AR
Large networks										
Simplebaseline	Resnet-50	256 × 192	34.0	8.9	70.0	90.9	77.9	66.8	75.8	75.6
RSN	3 × RSN50	256 × 192	15.0	4.9	74.8	89.9	82.4	71.2	81.1	80.2
HRNet	HRNet-W32	384 × 288	28.5	16.0	74.9	92.5	82.8	71.3	80.9	80.1
UDP	HRNet-W32	384 × 288	28.7	16.1	76.1	92.5	83.5	72.8	82.0	81.3
Small networks										
MobileNetV2 1 ×	MobileNetV2	384 × 288	9.8	3.3	66.8	90.0	74.0	62.6	73.3	72.3
ShuffleNetV2 1 ×	ShuffleNetV2	384 × 288	7.6	2.8	62.9	88.5	69.4	58.9	69.3	68.9
Small HRNet	HRNet-W18	384 × 288	1.3	1.2	55.2	85.8	61.4	51.7	61.2	61.5
Lite-HRNet	Lite-HRNet-18	384 × 288	1.1	0.4	66.9	89.4	74.4	64.0	72.2	72.6
	Lite-HRNet-30	384 × 288	1.8	0.7	69.7	90.7	77.5	66.9	75.0	75.4
Dite-HRNet	Dite-HRNet-18	384 × 288	1.1	0.4	68.4	89.9	75.8	65.2	73.8	74.4
	Dite-HRNet-30	384 × 288	1.8	0.7	70.6	90.8	78.2	67.4	76.1	76.4
MDW-HRNet(ours)	MDW-HRNet-18	384 × 288	1.1	0.4	69.0	90.1	75.9	65.8	75.2	74.7
	MDW-HRNet-30	384 × 288	1.8	0.7	70.9	91.0	78.3	67.6	77.3	76.8

**Figure 5:**
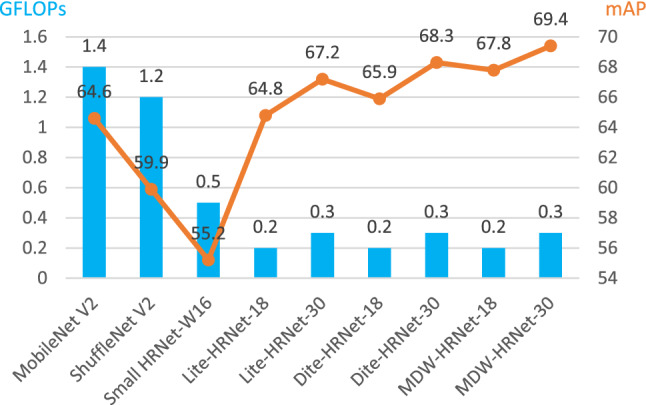
Comparison of complexity and accuracy for small networks.

For the results of the above types of experiments, MDW-HRNet shows higher accuracy compared to Lite-HRNet and Dite-HRNet with almost the same computational power consumption, and even the performance of MDW-HRNet-18 It is close to the case of Lite-HRNet-30, which is enough to show the state-of-the-art of our network structure. We publish a comparison of these small networks in Fig. 5,the results come from Table 2, the default input is 256 × 192.

#### MPII val

Table 4 shows the results of our network compared with other lightweight networks, here we set a single input image size of 256 × 256 for all networks. It can be seen that MDW-HRNet-18 improves PCKh by 0.9 points compared to Lite-HRNet-18 and 0.2 points compared to Dite-HRNet-18. Compared with MobileNetV2, MDW-HRNet-30 only consumes 18.1% of Params and 21% of GFLOPs, but improves the PCKh score by 2 points. Compared with MobileNetV3, ShuffleNetV2, and Small HRNet, MDW-HRNet also has advantages in speed and accuracy.

**Table 4:** Comparison in MPII val set.

Method	#Params	GFLOPs	PCKh
MobileNetV2 1 ×	9.6	1.9	85.4
MobileNetV3 1 × ^22^	8.7	1.8	84.3
ShuffleNetV2 1 ×	7.6	1.7	82.8
Small HRNet	1.3	0.7	80.2
Lite-HRNet-18	1.1	0.2	85.9
Lite-HRNet-30	1.8	0.4	86.9
Dite-HRNet-18	1.1	0.2	86.3
Dite-HRNet-30	1.8	0.4	87.2
MDW-HRNet-18	1.1	0.2	86.5
MDW-HRNet-30	1.8	0.4	87.4

### Ablation study

Our ablation experiments rely on the COCO dataset with a default input image size of 256 × 192.

#### Context information modeling and cross-channel dynamic convolution

**Figure 6:**
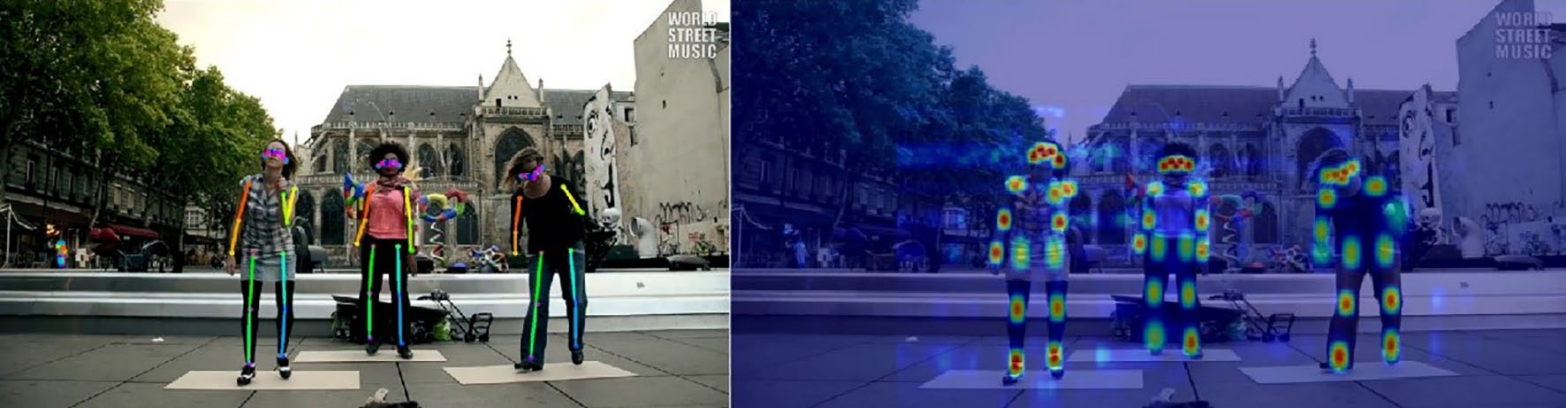
Visualization of pose estimation results of MDW-HRNet-18.

In order to verify the effectiveness of the context modeling module and cross-channel dynamic convolution proposed by us in MDW-HRNet, we conducted a series of ablation experiments in COCO val. First of all, we remove the CDC and GCM modules from the network structure, only retain the basic model structure, define this network as Simple MDW-HRNet-18, and on this basis, gradually add functional modules, and compare the results with the latest Lite-HRNet and Dite-HRNet. The results are shown in Table 5. On the basis of lightweight structure, the network can approach or even surpass the final training results of Lite-HRNet-18 only by relying on a single module. The context modeling or dynamic convolution proposed by us both provide higher gains for the network, but only increase minimal computing power requirements. In Fig. 6, we show the pose estimation visualization results of MDW-HRNet-18.

**Table 5:** Ablation experiments in COCO val set, where CDC stands for cross -channel dynamic convolution and GCM stands for Global Context Modeling.

Method	#Params(M)	COCO	
		MFLOPs	AP
Lite-HRNet-18	1.09	204.14	64.8
Dite-HRNet-18	1.19	209.80	65.9
Simple MDW-HRNet-18	0.91	186.03	64.4
Simple MDW-HRNet-18 (with CDC)	0.96	187.72	65.1
Simple MDW-HRNet-18 (with GCM)	1.12	206.48	66.4
MDW-HRNet-18	1.17	208.17	67.8

#### Hyperparameters in cross-channel dynamic convolution

The channel interaction in cross-channel dynamic convolution relies on one-dimensional convolution, and the number of convolution kernels K in one-dimensional convolution will affect the final performance of the network to a certain extent, in order to obtain the best effect, we have carried out a series of experimental studies on the value of K. On this basis, the specific value of the one-dimensional convolution K is analyzed, and the experimental results are shown in Table 6. The network gets the best training result when K = 3. It is worth mentioning that the value of K has little effect on the complexity of the network.

**Table 6:** The influence of the number of K on the training results.

Method	#Params	COCO				
	(M)	MFLOPs	AP	$AP^{50}$	$AP^{75}$	AR
MDW-HRNet-18 (K = 9)	1.17	208.17	67.2	89.4	74.8	71.6
MDW-HRNet-18 (K = 7)	1.17	208.17	67.6	89.5	76.0	71.9
MDW-HRNet-18 (K = 5)	1.17	208.17	67.1	89.4	74.9	71.6
MDW-HRNet-18 (K = 3)	1.17	208.17	67.8	90.5	76.0	72.2

#### Hyperparameters in cross-channel dynamic convolution

In this section, we replace the cross-channel dynamic convolution in the network with other dynamic convolutions and carry out experiments, including condconv, dynamicconv, ODConv, to verify the performance gain of CDC in the attitude estimation task. The experimental results are shown in Table 7. The resource consumption of cross-channel dynamic convolution is close to that of other dynamic convolutions, and the highest accuracy is achieved.

**Table 7:** Comparison between CDC and other types of dynamic convolution.

Method	#Params(M)	COCO	
		MFLOPs	AP
MDW-HRNet-18 (without CDC)	1.12	206.48	66.43
MDW-HRNet-18	1.17	208.17	67.79
MDW-HRNet-18 (with Dynamic_conv)	1.16	210.51	67.33
MDW-HRNet-18 (with ODConv)	1.22	213.56	67.20
MDW-HRNet-18 (with CondConv)	1.21	211.52	67.17

In Table 7, MDW-HRNet-18 (without CDC) means that the network has removed the CDC module, and MDW-HRNet-18 (with Dynamic_conv) means that the network uses Dynamic Conv replaces CDC, and so on.

## Conclusion and discussion

In order to solve the problem that the scale of extracted feature weights in the lightweight pose estimation network is relatively single and lacks sufficient information interaction channels, we propose an improved multi-dimensional weighted high-resolution network named MDW-HRnet, which has achieved good experimental results in COCO and MPII data sets, surpassing The current mainstream small pose estimation network. The performance improvement depends on the following aspects: (i) The new structure retains the advantages of high-resolution networks while further optimizing the way of feature fusion; (ii) Cross-channel dynamic convolution is proposed, which will further facilitate accurate keypoint localization. (iii) Multi-dimensional weighted convolution module is proposed, including spatial weighting, channel weighting and convolution weighting, so as to further mine performance. It is worth mentioning that although our method has achieved good results in lightweight networks, the performance of the network still has a certain gap compared with large networks. In the next step, we will further verify the effect of the proposed performance module on other tasks, and focus on further performance improvement.

## Data Availability

Data or code presented in this study is available on request from the corresponding author.

## References

[1] Cai, Y., Wang, Z., Luo, Z. *et al*. Learning delicate local representations for multi-person pose estimation. In *European Conference on Computer Vision*. Springer. 455–472 (2020).

[2] Sun, K., Xiao, B., Liu, D. *et al*. Deep high-resolution representation learning for human pose estimation. In *Proceedings of the IEEE/CVF Conference on Computer Vision and Pattern Recognition*. 5693–5703 (2019).

[3] Cheng, B., Xiao, B., Wang, J. *et al.* Higherhrnet: Scale-aware representation learning for bottom-up human pose estimation. In *Proceedings of the IEEE/CVF Conference on Computer Vision and Pattern Recognition*. 5386–5395 (2020).

[4] Geng, Z., Sun, K., Xiao, B. *et al.* Bottom-up human pose estimation via disentangled keypoint regression. In *Proceedings of the IEEE/CVF Conference on Computer Vision and Pattern Recognition*. 14676–14686 (2021).

[5] Howard, A., Sandler, M., Chu, G. *et al*. Searching for mobilenetv3. In *Proceedings of the IEEE/CVF International Conference on Computer Vision*. 1314–1324 (2019).

[6] Howard, A. G., Zhu, M., Chen, B. *et al*. Mobilenets: Efficient convolutional neural networks for mobile vision applications. arXiv preprint arXiv:1704.04861 (2017).

[7] Ma, N., Zhang, X., Zheng, H. T. *et al*. Shufflenet v2: Practical guidelines for efficient cnn architecture design. In *Proceedings of the European conference on computer vision (ECCV)*. 116–131 (2018).

[8] Zhang, X., Zhou, X., Lin, M. *et al*. Shufflenet: An extremely efficient convolutional neural network for mobile devices. In *Proceedings of the IEEE Conference on Computer Vision and Pattern Recognition*. 6848–6856 (2018).

[9] Yu, C., Xiao, B., Gao, C. *et al.* Lite-hrnet: A lightweight high-resolution network. In *Proceedings of the IEEE/CVF Conference on Computer Vision and Pattern Recognition*. 10440–10450 (2021).

[10] Li, Q., Zhang, Z., Xiao, F. *et al*. Dite-HRNet: Dynamic lightweight high-resolution network for human pose estimation. arXiv preprint arXiv:2204.10762 (2022).

[11] Li, W., Wang, Z., Yin, B. *et al*. Rethinking on multi-stage networks for human pose estimation. arXiv preprint arXiv:1901.00148 (2019).

[12] Ou Z, Luo YM, Chen J (2022). SRFNet: Selective receptive field network for human pose estimation. J. Supercomput..

[13] Yang, W., Li, S., Ouyang, W. *et al*. Learning feature pyramids for human pose estimation. In *Proceedings of the IEEE International Conference on Computer Vision*. 1281–1290 (2017).

[14] Zhang Z, Luo Y, Gou J (2021). Double anchor embedding for accurate multi-person 2D pose estimation. Image Vis. Comput..

[15] Guo MH, Liu ZN, Mu TJ (2022). Beyond self-attention: External attention using two linear layers for visual tasks. IEEE Trans. Pattern Anal. Mach. Intell..

[16] Hou, Q., Zhou, D., Feng, J. Coordinate attention for efficient mobile network design. In *Proceedings of the IEEE/CVF conference on computer vision and pattern recognition*. 13713–13722 (2021).

[17] Zhang, H., Zu, K., Lu, J. *et al*. EPSANet: An efficient pyramid squeeze attention block on convolutional neural network. In *Proceedings of the Asian Conference on Computer Vision*. 1161–1177 (2022).

[18] Hu, J., Shen, L., Sun, G. Squeeze-and-excitation networks. In *Proceedings of the IEEE conference on computer vision and pattern recognition*. 7132–7141 (2018).

[19] Tan, M., Le, Q. V. Mixconv: Mixed depthwise convolutional kernels. arXiv preprint arXiv:1907.09595, (2019).

[20] He, K., Zhang, X., Ren, S. *et al*. Deep residual learning for image recognition. In *Proceedings of the IEEE Conference on Computer Vision and Pattern Recognition*. 770–778 (2016).

[21] Liu, Z., Mao, H., Wu, C. Y. *et al*. A convnet for the 2020s. In *Proceedings of the IEEE/CVF Conference on Computer Vision and Pattern Recognition*. 11976–11986 (2022).

[22] Yang, B., Bender, G., Le, Q. V., *et al*. Condconv: Conditionally parameterized convolutions for efficient inference. In *Advances in Neural Information Processing Systems*. 32 (2019).

[23] Chen, Y., Dai, X., Liu, M. *et al*. Dynamic convolution: Attention over convolution kernels. In *Proceedings of the IEEE/CVF Conference on Computer Vision and Pattern Recognition*. 11030–11039 (2020).

[24] Li, C., Zhou, A., Yao, A. Omni-dimensional dynamic convolution. arXiv preprint arXiv:2209.07947, (2022).

[25] Zhu Z, Xu M, Bai S, et al. Asymmetric non-local neural networks for semantic segmentation. In *Proceedings of the IEEE/CVF International Conference on Computer Vision*. 593–602 (2019).

[26] Cao, Y., Xu, J., Lin, S. *et al*. Gcnet: Non-local networks meet squeeze-excitation networks and beyond. In *Proceedings of the IEEE/CVF International Conference on Computer Vision Workshops*. 0–0 (2019).

[27] Fu, J., Liu, J., Tian, H. *et al*. Dual attention network for scene segmentation. In *Proceedings of the IEEE/CVF Conference on Computer Vision and Pattern Recognition*. 3146–3154 (2019).

[28] Liu, H., Liu, F., Fan, X., *et al*. Polarized self-attention: Towards high-quality pixel-wise regression. arXiv preprint arXiv:2107.00782, (2021).

[29] Chen, Y., Wang, Z., Peng, Y. *et al*. Cascaded pyramid network for multi-person pose estimation. In *Proceedings of the IEEE Conference on Computer Vision and Pattern Recognition*. 7103–7112 (2018).

[30] Luo Y, Ou Z, Wan T (2022). FastNet: Fast high-resolution network for human pose estimation. Image Vis. Comput..

[31] Xu, Y., Zhang, J., Zhang, Q. *et al*. ViTPose: Simple vision transformer baselines for human pose estimation. arXiv preprint arXiv:2204.12484, (2022).

[32] Wang, Q., Wu, B., Zhu, P. *et al.* ECA-Net: Efficient channel attention for deep convolutional neural networks. In *Proceedings of the IEEE/CVF Conference on Computer Vision and Pattern Recognition*. 11534–11542 (2020).

[33] Lin, T. Y., Maire, M., Belongie, S. *et al*. Microsoft coco: Common objects in context. In *European Conference on Computer Vision*. Springer, Cham. 740–755 (2014).

[34] Andriluka, M., Pishchulin, L., Gehler, P. *et al*. 2d human pose estimation: New benchmark and state of the art analysis. In *Proceedings of the IEEE Conference on Computer Vision and Pattern Recognition*. 3686–3693 (2014).

[35] Xiao, B., Wu, H., Wei, Y. Simple baselines for human pose estimation and tracking. In *Proceedings of the European Conference on Computer Vision (ECCV)*. 466–481 (2018).

[36] Newell, A., Yang, K., Deng, J. Stacked hourglass networks for human pose estimation. In *European Conference on Computer Vision*. Springer. 483–499 (2016).

